# Supplementary material for: A machine learning-based approach for constructing remote photoplethysmogram signals from video cameras
Source: Commun Med (Lond). 2024 Jun 7;4:109. doi: 10.1038/s43856-024-00519-6 (PMC11161609; doi:10.1038/s43856-024-00519-6)
Supplement: Supplementary file 2 — SUPPLEMENTAL MATERIAL [file 43856_2024_519_MOESM2_ESM.pdf]

Supplementary information: Results and p-values for the datasets PURE, LGI-PPGI and MR-NIRP

Supplementary Table 1. Comparison across datasets results for different metrics. Our model outperforms the other models, especially in the case of the metric DTW, but also for the dataset PURE, our model is better than the other methods for all the metrics.

		DTW	r	RMSE	$\|\Delta HR\|$
PURE	GREEN	2.88	0.66	0.4	2.02
	LGI	2.3	0.72	0.46	0.57
	CHROM	1.72	0.73	0.2	0.58
	POS	2.28	0.72	0.46	0.53
	GRGB	2.59	0.69	0.41	0.7
	Our Model	1.17	0.83	0.2	0.52
LGI-PPGI	GREEN	3.2	0.4	0.37	16.09
	LGI	2.89	0.48	0.38	7.9
	CHROM	2.69	0.47	0.26	9.13
	POS	2.84	0.51	0.38	5.07
	GRGB	2.98	0.43	0.36	8.99
	Our Model	1.65	0.49	0.31	6.15
MR-NIRP	GREEN	3.4	0.22	0.31	10.86
	LGI	2.97	0.21	0.31	7.81
	CHROM	2.98	0.22	0.31	7.73
	POS	2.87	0.23	0.31	7.53
	GRGB	3.1	0.2	0.31	11.27
	Our Model	1.73	0.24	0.39	7.45

Supplementary Table 2. The p-values for all the metrics across datasets. In most of the metrics the differences are significant, except for RMSE and $|\Delta HR|$, where the differences between our Model, CHROM and POS are non-significant. The p-values are obtained by applying the Friedman and post hoc Nemenyi tests.

		DTW	r	RMSE	$\|\Delta HR\|$
PURE	GREEN vs. Our Model	0.001	0.001	0.001	0.001
	LGI vs. Our Model	0.001	0.001	0.001	0.9
	CHROM vs. Our Model	0.16	0.01	0.9	0.864
	POS vs. Our Model	0.001	0.001	0.001	0.9
	GRGB vs. Our Model	0.001	0.001	0.001	0.01
LGI-PPGI	GREEN vs. Our Model	0.001	0.001	0.9	0.001
	LGI vs. Our Model	0.001	0.9	0.671	0.195
	CHROM vs. Our Model	0.009	0.9	0.034	0.027
	POS vs. Our Model	0.003	0.811	0.811	0.9
	GRGB vs. Our Model	0.001	0.007	0.9	0.003
MR-NIRP	GREEN vs. Our Model	0.001	0.664	0.001	0.003
	LGI vs. Our Model	0.006	0.263	0.053	0.9
	CHROM vs. Our Model	0.001	0.664	0.001	0.9
	POS vs. Our Model	0.114	0.9	0.142	0.9
	GRGB vs. Our Model	0.001	0.008	0.04	0.217

Supplementary Table 3. Comparison across activities results for different metrics. Our model outperforms the rest of the models in most of the activities and metrics.

		DTW	r	RMSE	$\|\Delta HR\|$
Rest	GREEN	2.82	0.52	0.38	4.25
	LGI	2.53	0.52	0.4	3.5
	CHROM	2.2	0.52	0.24	3.81
	POS	2.46	0.53	0.41	3.54
	GRGB	2.69	0.5	0.38	3.82
	Our Model	1.45	0.57	0.29	3.51
Talk	GREEN	3.28	0.34	0.34	12.92
	LGI	2.83	0.4	0.36	6.31
	CHROM	2.68	0.42	0.27	6.19
	POS	2.81	0.42	0.36	4.86
	GRGB	3.02	0.38	0.34	8.94
	Our Model	1.58	0.46	0.32	5.38
Translation	GREEN	2.56	0.72	0.43	1.09
	LGI	2.2	0.74	0.47	0.31
	CHROM	1.58	0.75	0.19	0.38
	POS	2.2	0.74	0.47	0.33
	GRGB	2.46	0.72	0.43	0.46
	Our Model	1.2	0.84	0.19	0.33
Rotation	GREEN	3.38	0.59	0.36	4.46
	LGI	2.56	0.69	0.44	1.77
	CHROM	2.03	0.71	0.2	1.71
	POS	2.5	0.71	0.44	2.01
	GRGB	2.86	0.65	0.39	2.6
	Our Model	1.18	0.77	0.2	1.65
Gym	GREEN	3.17	0.24	0.34	32
	LGI	2.8	0.32	0.34	14.93
	CHROM	2.83	0.28	0.32	20.49
	POS	2.79	0.42	0.34	3.71
	GRGB	2.8	0.31	0.34	14.38
	Our Model	2.02	0.4	0.36	8.84

Supplementary Table 4. The p-values for all the metrics across activities. The differences between our model and the other methods are significant in most of the metrics, except for $|\Delta HR|$. The p-values are obtained by applying the Friedman and post hoc Nemenyi tests.

		DTW	r	RMSE	$\|\Delta HR\|$
Rest	GREEN vs. Our Model	0.001	0.118	0.9	0.118
	LGI vs. Our Model	0.001	0.169	0.459	0.9
	CHROM vs. Our Model	0.064	0.459	0.169	0.9
	POS vs. Our Model	0.004	0.9	0.052	0.9
	GRGB vs. Our Model	0.001	0.001	0.9	0.9
Talk	GREEN vs. Our Model	0.001	0.001	0.9	0.001
	LGI vs. Our Model	0.001	0.009	0.842	0.509
	CHROM vs. Our Model	0.007	0.652	0.045	0.307
	POS vs. Our Model	0.004	0.9	0.9	0.9
	GRGB vs. Our Model	0.001	0.001	0.9	0.004
Translation	GREEN vs. Our Model	0.001	0.001	0.005	0.001
	LGI vs. Our Model	0.002	0.009	0.001	0.9
	CHROM vs. Our Model	0.892	0.268	0.9	0.789
	POS vs. Our Model	0.004	0.013	0.001	0.9
	GRGB vs. Our Model	0.001	0.001	0.017	0.113
Rotation	GREEN vs. Our Model	0.001	0.001	0.025	0.001
	LGI vs. Our Model	0.001	0.009	0.001	0.64
	CHROM vs. Our Model	0.257	0.9	0.9	0.9
	POS vs. Our Model	0.001	0.159	0.001	0.9
	GRGB vs. Our Model	0.001	0.001	0.001	0.017
Gym	GREEN vs. Our Model	0.002	0.03	0.489	0.017
	LGI vs. Our Model	0.41	0.745	0.489	0.745
	CHROM vs. Our Model	0.207	0.087	0.489	0.41
	POS vs. Our Model	0.636	0.9	0.489	0.9
	GRGB vs. Our Model	0.41	0.41	0.489	0.527
